# Clinical daily rhythms of seizure in different subtypes of temporal lobe epilepsy

**DOI:** 10.3389/fneur.2025.1599236

**Published:** 2025-05-14

**Authors:** Shuxian Gong, Zhongyuan Long, Dongyan Ji, Qiang Guo, Tianpeng Zhang, Shaochun Li, Xiaofeng Yang, Lisen Sui

**Affiliations:** ^1^The Second Clinical Medical College of Guangzhou University of Chinese Medicine, Guangzhou, China; ^2^Department of Neurosurgery, Guangdong Sanjiu Brain Hospital, Guangzhou, China; ^3^Institute of Molecular Rhythm and Metabolism, Guangzhou University of Chinese Medicine, Guangzhou, China; ^4^Guangzhou National Laboratory, Guangzhou, Guangdong, China; ^5^The First Affiliated Hospital, Guangzhou Medical University, Guangzhou, Guangdong, China

**Keywords:** temporal lobe epilepsy, rhythm, seizure frequency, seizure duration, clinical subtypes

## Abstract

**Introduction:**

The relationship between circadian rhythms and seizures in temporal lobe epilepsy (TLE) has been well recognized, but it remains poorly understood how the interaction between the endogenous clock system and seizures may affect seizure patterns and patient management. This study investigates the circadian rhythm patterns of clinical seizures in TLE, with a focus on different subtypes and clinical variables.

**Methods:**

We retrospectively analyzed the seizure rhythms of patients diagnosed with TLE who were admitted to the video-EEG ward. Patients were categorized based on clinical data, including mesial, lateral, mesio-lateral, and temporal pole types, as well as left, right, and bilateral temporal lobe involvement. Seizure onset times, frequency, and duration were recorded for each patient.

**Results:**

Our findings indicate that TLE patients exhibit notable seizure rhythms, with the peak times of seizure frequency and duration differing across subtypes and age groups. Notably, patients with mesial and mesio-lateral temporal lobe epilepsy showed peak seizure durations between 23:00 and 24:00, while seizure frequency peaked between 19:00 and 20:00 for right temporal lobe patients and between 19:00 and 22:00 for left temporal lobe patients. Additionally, children and adults had distinct seizure rhythms, with children peaking between 21:00 and 24:00, while adults had a peak frequency between 19:00 and 22:00.

**Discussion:**

These findings highlight the variability of seizure rhythms in TLE and underscore the need for personalized treatment strategies that consider circadian factors, potentially leading to better seizure management and therapeutic interventions.

## 1 Introduction

Epilepsy is a chronic brain disorder characterized by recurrent, paroxysmal, and transient central nervous system dysfunction resulting from the abnormal excessive discharge of neurons in the brain. According to World Health Organization (WHO) statistics, approximately 50 million people worldwide are affected by epilepsy, with nearly one-third classified as having refractory epilepsy. In China, there are around 10 million individuals with epilepsy, of whom 30–35% have temporal lobe epilepsy (TLE). Drug therapy is the first-line treatment for TLE, and most patients can achieve effective seizure control with anti-seizure medications (ASMs). However, over 30% of patients either resist existing medications or develop drug-resistant epilepsy due to delayed treatment, with the emergence of drug resistance to conventional ASMs presenting significant challenges to effective management. In clinical practice, only a subset of drug-resistant TLE cases can be managed through surgical interventions, such as temporal lobe resection, selective amygdalohippocampectomy, or stereotactic electroencephalography-guided radiofrequency thermocoagulation. Therefore, it is essential to explore more optimized treatment strategies for TLE to enhance efficacy and minimize side effects for a larger population of TLE patients (1, 2).

In clinical practice, the unpredictability of epileptic seizures is a prominent characteristic that poses significant challenges for treatment. Recently, chronopharmacology has emerged as a promising treatment approach aimed at enhancing clinical effectiveness and tolerability. Tailoring treatment according to the rhythmicity of diseases can markedly improve drug efficacy while minimizing toxicity and side effects. This therapeutic strategy has been applied across various medical conditions (3–5). Over the centuries, the rhythmicity of epileptic seizures has gradually gained recognition, with different types of epilepsy exhibiting specific circadian patterns in seizure occurrence. Notably, clinical seizures in patients with TLE also display distinct circadian rhythms. For instance, Pavlova et al. reported that 50% of seizure occurrences in 15 TLE patients transpired between 15:00 and 19:00 (6). Similarly, Karafin et al. identified that the peak times for clinical seizures in 60 patients with mesial temporal lobe epilepsy were between 7:00 and 8:00, as well as between 16:00 and 17:00 (7). Furthermore, Durazzo et al. documented a bimodal distribution of TLE seizures in 45 patients, with a primary peak occurring between 16:00 and 19:00 and a secondary peak between 7:00 and 10:00 (8). Previous studies have indicated that peak seizure occurrences generally occur between 13:00 and 16:00. Latini et al. found that patients with left temporal lobe epilepsy (LTLE) exhibited a heightened rate of seizures between 08:00 and 16:00, whereas those with right temporal lobe epilepsy (RTLE) experienced peak seizure rates between 12:00 and 16:00 (9). This variability in seizure rhythm patterns among different researchers suggests that clinical variables, including demographics, subtypes of temporal lobe epilepsy, and the laterality of seizure onset as observed in scalp electroencephalography, may play a significant role (7). TLE can be categorized according to the Kahane classification into several types: mesial temporal lobe, lateral temporal lobe, mesio-lateral, polar, and temporal plus (10). Additionally, based on the laterality of the lesion and the origin of scalp electroencephalographic seizures, TLE can be further classified as left temporal, right temporal, or bilateral (9). Consequently, identifying clinical seizure rhythm patterns across different TLE subtypes is essential for developing chronotherapy strategies and enhancing treatment outcomes for patients. In this study, we performed a retrospective analysis of video long-term electroencephalographic data from 213 patients with refractory TLE. We assessed the frequency and duration of seizures during their electroencephalographic monitoring period utilizing both the Kahane classification and the classification based on the origin of scalp electroencephalographic seizures. The objective was to elucidate the daily rhythms of seizures in various TLE subtypes, thereby facilitating more targeted treatment approaches for patients with TLE. In summary, this study reveals the seizure rhythm patterns across different temporal lobe epilepsy subtypes. Our findings offer new insights for the clinical treatment of TLE and lay a novel foundation for the long-term management of TLE patients.

## 2 Methods

All patients were hospitalized in the Epilepsy Center of Guangdong Provincial Hospital of Traditional Chinese Medicine and the 8th and 1st Departments of Neurosurgery of Guangdong Sanjiu Brain Hospital. This retrospective study has been approved by the Ethics Office of Guangdong Provincial Hospital of Traditional Chinese Medicine, the leader of the research team. Inclusion criteria are long-distance video EEG monitoring of epilepsy patients with seizures during hospitalization (11–13). Exclusion criteria are patients with incomplete clinical data. Therefore, 213 patients were included in the analysis.

### 2.1 Diagnosis

Clinical and electrographic features were analyzed through video long-term electroencephalography (VEEG) recordings, conducted over a period exceeding 24 h. Seizure events were evaluated by two experts proficient in VEEG analysis, with each seizure examined a minimum of five times to ascertain the electroclinical characteristics (14, 15). All patients underwent evaluation using at least a 3.0 T MRI with an epilepsy-specific protocol. The epileptogenic zone was determined by more than two specialized epileptologists, based on the patient’s clinical symptomatology, electroencephalographic analysis, and MRI epilepsy sequences (11, 12). TLE patients were classified according to the Kahane classification into categories such as mesial temporal lobe, lateral temporal lobe, mesio-lateral, polar temporal, and temporal plus. Based on the lateralization of seizure onset observed during VEEG monitoring, patients were categorized as left temporal, right temporal, or bilateral. Patients for whom the laterality and localization of seizure events could not be established were excluded, as were those with non-epileptic events.

### 2.2 Video electroencephalography

The Nihon Kohden Neurofax EEG-1200 EEG system was utilized to record electroencephalography (EEG) data, with electrodes positioned on the scalp in accordance with the international 10–20 system. A sampling frequency of 1,000 Hz was employed, and a total of 32 electrodes were affixed to the scalp. Continuous VEEG monitoring was conducted for all patients, with VEEG records obtained throughout the monitoring period. This VEEG monitoring extended for 24 h each day and was overseen and archived by trained EEG technicians. If deemed necessary, antiepileptic medications were gradually tapered beginning on the first day of recording. Additionally, family members or caregivers were encouraged to activate the seizure alarm in the event of seizure occurrences.

### 2.3 Video electroencephalography data

The variables analyzed from the video electroencephalography recordings included the total number of seizures, seizure frequency, seizure duration, and the count of seizures occurring during wakefulness and sleep for each subtype, assessed in two-hour intervals (01:00–02:00, 03:00–04:00, 05:00–06:00, 07:00–08:00, 09:00–10:00, 11:00–12:00, 13:00–14:00, 15:00–16:00, 17:00–18:00, 19:00–20:00, 21:00–22:00, 23:00–24:00). Additionally, the duration of seizures occurring during both wakefulness and sleep was recorded. The classification of wakefulness and sleep periods adhered to the standards set by the American Academy of Sleep Medicine and was further refined through video analysis of the EEG signal.

### 2.4 Statistical analysis

All data were normalized on a 24-h cycle, and inter-group analysis was performed based on clinical variables such as patient type, age, gender, etc. Before curve fitting, the Mean ± SEM of each group was obtained and the peak time points of each group were observed. This part of the analysis was performed in GraphPad Prism 8.0.2. We used the JTK-CYCLE algorithm to perform rhythm analysis on the collected onset time points and durations (16). JTK_CYCLE is a nonparametric rank-based test. The algorithm was implemented by R version 4.4.1.^1^ Our goal is to discover the rhythmicity of seizure frequency and seizure duration in each subject within a standardized 24 h, focusing on Adjusted Parameter (ADJ.P) < 0.05 and Benjamani-Hochberg q-value (BH.Q < 0.05) (17). Given that our sampling interval is 2 h, a total of 12 time points, repeated once, our JTK-CYCLE code configuration is as follows: jtkdist (ncol(data), reps = 1), periods < −10:14, jtk.init (periods, 2). The remaining parameters remain default. Demographic data analysis was completed using SPSS version 25.0 (SPSS Inc., Chicago, IL, USA).

## 3 Results

### 3.1 Demographics

Of the 213 participants, 115 (54%) were male and 98 (46%) were female. There were 79 participants (37.1%) of the mesial temporal lobe type, 26 participants (12.2%) of the lateral temporal lobe type, 25 participants with mesio-lateral temporal lobe (11.7%), 9 participants (4.2%) with temporal polar type, and 74 participants (34.7%) with temporal plus type. 109 participants (51.2%) had LTLE, 92 participants (43.2%) had RTLE, and 12 participants (5.6%) had bilateral temporal lobe epilepsy. In the age group, 44 participants (20.7%) were under 18 years old, and 168 participants (78.9%) were between 18 and 65 years old (Tables 1, 2).

**Table 1:** Statistical analysis of 24-h seizure duration and frequency in TLE under different categories by JTK.Cycle.

JTK.Cycle result	Clinical variables	Mesial (*n* = 79)	Lateral (*n* = 26)	Mesio-lateral (*n* = 25)	Temporo-polar (*n* = 9)	Plus (*n* = 74)	Female (*n* = 98)	Male (*n* = 115)	<18 (*n* = 44)	18–65 (*n* = 168)	>65 (*n* = 1)	Right (*n* = 92)	Left (*n* = 109)	Bilateral (*n* = 12)	Total (*n* = 213)
ADJ.P	Frequency	0.15	0.57	*0.03	0.15	0.15	0.15	1	*0.01	**0.002	NA	*0.01	*0.03	*0.03	***<0.001
	Duration	***<0.001	1	**0.01	0.15	1	0.15	0.15	1	0.1	NA	0.57	0.57	***<0.001	***<0.001
BH.Q	Frequency	0.150	1	*0.03	0.150	0.30	0.150	1	*0.026	**0.004	NA	*0.026	0.06	*0.031	***<0.001
	Duration	***<0.001	1	*0.026	0.150	1	0.150	0.30	1	0.10	NA	0.572	0.57	***0.001	***<0.001
PER	Frequency	26	24	24	24	24	24	26	24	24	NA	24	24	24	22
	Duration	24	24	24	22	26	22	24	26	22	NA	24	24	26	24
AMP	Frequency	0.073	0.043	0.097	0.105	0.070	0.105	0.073	0.054	0.079	NA	0.038	0.038	0.313	0.065
	Duration	12.488	16.789	16.193	7.637	5.119	7.637	3.408	8.222	5.471	NA	3.499	3.781	27.323	5.415

Adjusted Parameter *p*-value (ADJ.P) <0.05 indicates that the data is statistically significant and has circadian rhythm. Amplitude (AMP): Lower amplitude indicates that the signal changes relatively mildly, not particularly strongly. This may mean that changes in the biological process or gene expression being examined are modest. Period (PER): A complete 24-h cycle coincides with the biological rhythm, suggesting that the frequency may be regulated by the circadian rhythm. Benjamani-Hochberg q-value (BH. Q): BH. Q < 0.05 strongly indicates that this is not a random fluctuation, but a true circadian signal with statistical significance. Bold values indicate statistical significance. *represents statistically significant differences. **indicates very significant statistical differences.***denotes highly significant statistical differences.

**Table 2:** Demographic characteristics classified by age, gender, Kahane classification, and laterality.

Characteristics	<18				>65				18–65				Total			
	N	Mean	Media	Range	N	Mean	Media	Range	N	Mean	Media	Range	N	Mean	Media	Range
Total	44	12.80	14.00	3, 17	1	66.00	66.00	66	168	32.08	30.00	18, 58	213	28.25	27.00	3, 66
Lateral	5	14.80	15.00	12, 17	0				21	32.62	29.00	20, 58	26	29.19	27.00	12, 58
Mesial	11	11.55	14.50	3, 15	1	66.00	66.00	66	67	32.43	30.00	18, 58	79	29.95	28.00	3, 66
Mesio-lateral	5	13.40	12.00	10, 17	0				20	30.70	29.00	18, 57	25	27.24	24.00	10, 57
Temporo-plus	21	13.24	14.00	8, 17	0				53	31.60	30.00	18, 58	74	26.39	25.50	8, 58
Temporo-polar	2	8.50	8.50	5, 12	0				7	34.57	32.00	21, 55	9	28.78	27.00	5, 55
Bilateral	1	6.00	6.00	6	0				11	37.45	37.00	24, 55	12	34.83	35.50	6, 55
Left	27	13.44	14.00	3, 17	0				81	31.07	30.00	18, 55	109	27.03	27.00	3, 66
Right	16	12.13	12.00	5, 17	0				76	32.37	29.50	18, 58	92	28.85	27.00	5, 58
Female	22	12.23	13.00	3, 17	1	66.00	66.00	66	75	33.79	31.00	18, 58	98	29.28	27.50	3, 66
Male	22	13.36	14.00	6, 17	0				93	30.37	29.00	18, 57	115	27.38	27.00	6, 57

### 3.2 Patients with temporal lobe epilepsy present clinically distinct epileptic rhythms

**Figure 1:**
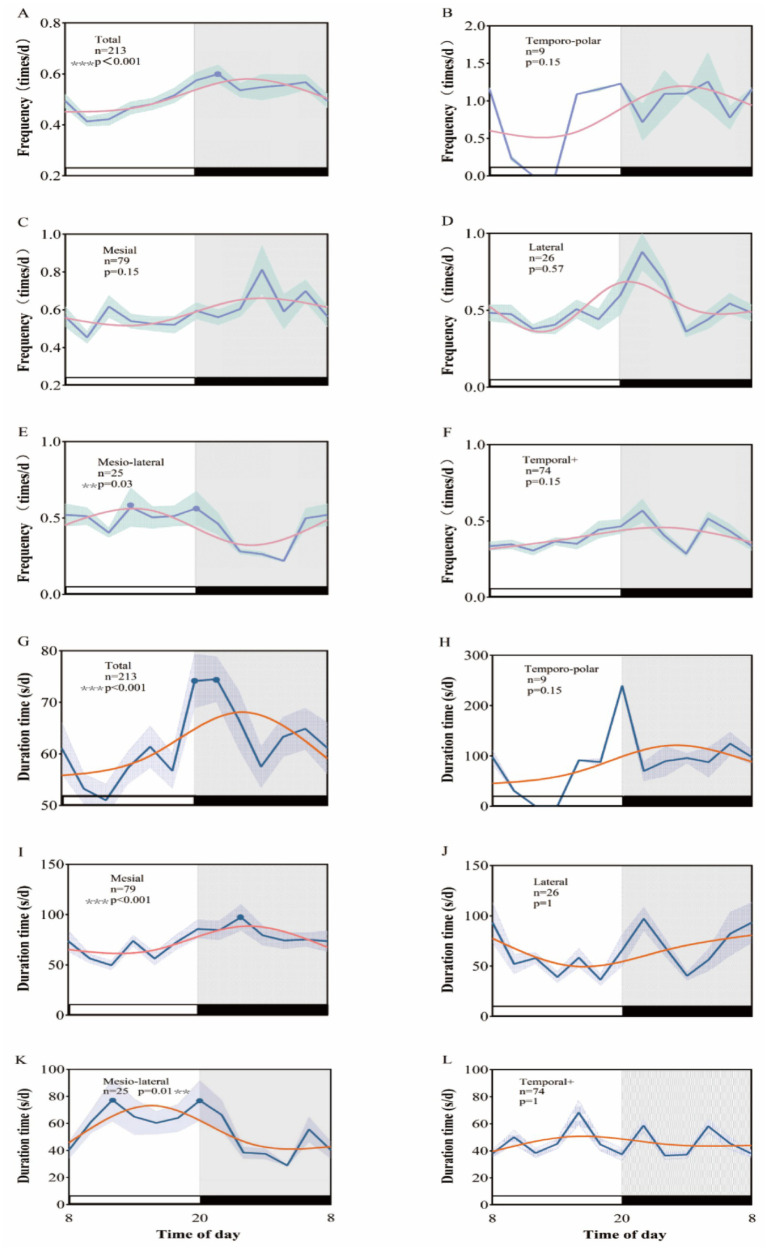
Rhythm patterns of clinical seizures in different subtypes of temporal lobe epilepsy after Kahane classification. A 24-h day is divided into 12 2-h intervals. The x-axis represents the start time of each bin. **(A–F)** Shows the rhythmic pattern of seizure frequency distribution, and **(G–L)** shows the rhythmic pattern of seizure duration.

**Figure 2:**
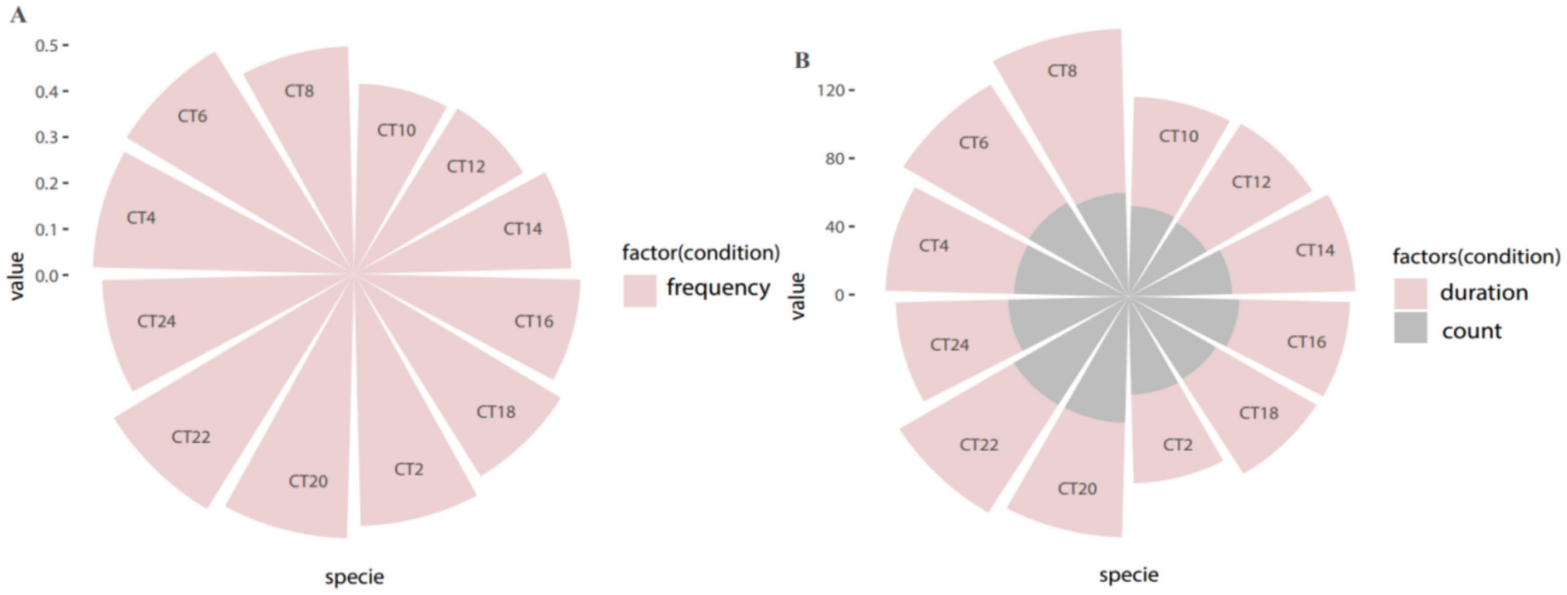
Period distribution of overall circadian rhythm in all patients. **(A)** Periodic distribution of attack frequency; **(B)** Periodic distribution of attack duration and number of attacks.

In this study involving 213 enrolled patients, we analyzed clinical seizure frequency and seizure duration (*p* < 0.001, *Q* < 0.001) using the rhythm analysis algorithm JTK-CYCLE (Table 1). Our findings indicate that the peak period of clinical seizures in TLE patients is concentrated between 19:00 and 22:00 (Figures 1, 2).

### 3.3 According to the Kahane classification, patients with mesial temporal lobe and mesio-lateral temporal lobe have clear clinical seizure rhythm

Kahane’s typology categorizes temporal lobe epilepsy into five distinct types: mesial temporal lobe, lateral temporal lobe, mesio-lateral temporal lobe, temporal polar, and temporal lobe plus. Analysis using the rhythm algorithm JTK-CYCLE revealed that the peak clinical seizure duration for patients with mesial temporal lobe epilepsy occurred between 23:00 and 24:00 (*p* < 0.01), while the frequency of clinical seizures did not exhibit a significant peak (*p* = 0.15). In patients with lateral temporal lobe epilepsy, neither the frequency of clinical seizures (*p* = 0.57) nor seizure duration (*p* = 1) displayed a biological rhythm. For those with mesio-lateral temporal lobe epilepsy, clinical seizure frequency demonstrated inconsistent circadian rhythm characteristics, with peaks occurring at 13:00–14:00 and 19:00–20:00 (*p* = 0.03), while peak seizure duration was noted at 11:00–12:00 and 19:00–20:00 (*p* = 0.01). Conversely, the analysis of EEG data for the temporal polar and temporal lobe plus epilepsy subtypes indicated no circadian rhythmic variations in either clinical seizure frequency (*p* = 0.15 for both) or clinical seizure duration (*p* = 0.15, *p* = 1, respectively) (Figure 1).

### 3.4 Clinical seizure rhythms are inconsistent across age groups

**Figure 3:**
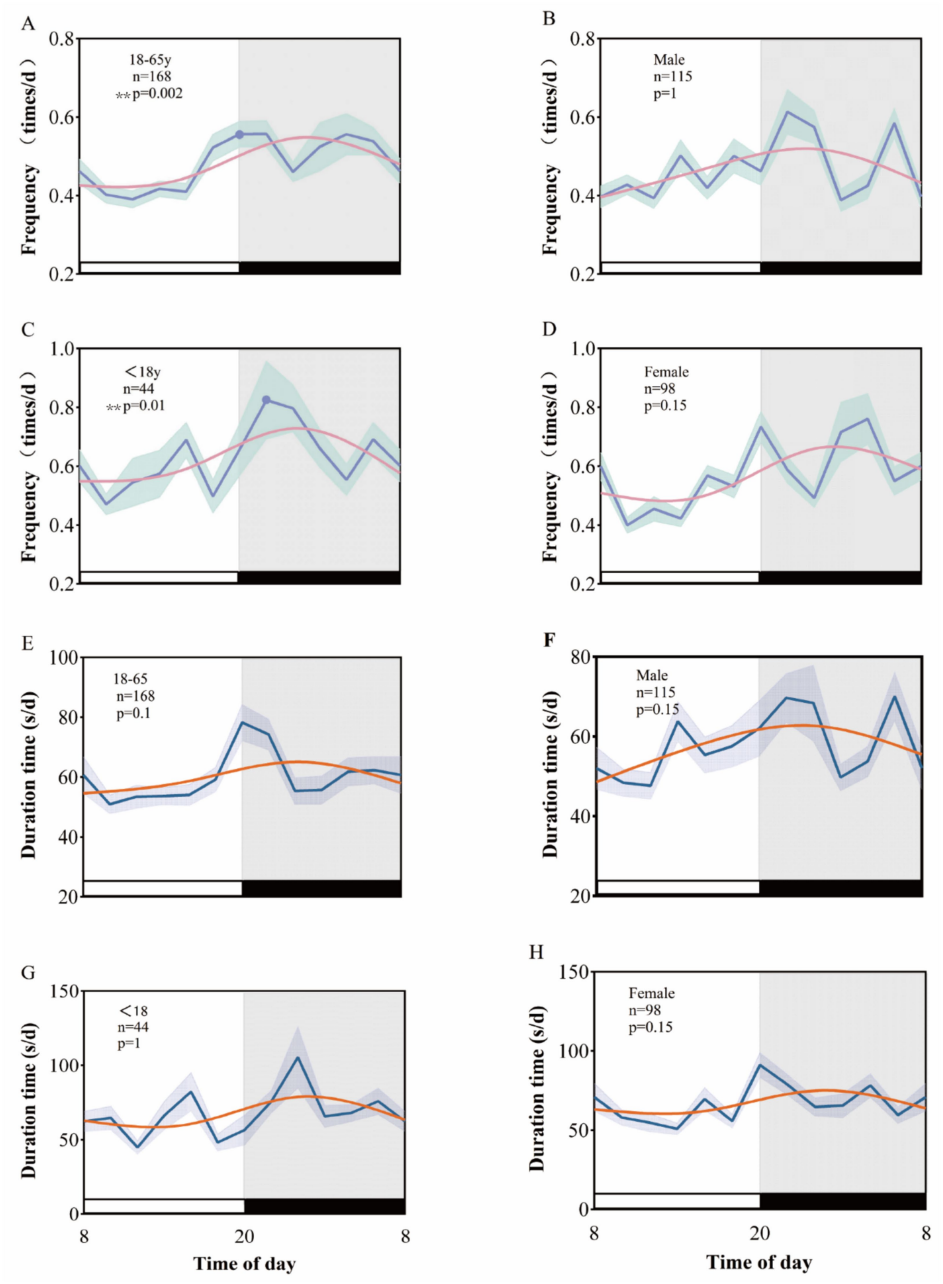
Rhythmic patterns of clinical seizures in temporal lobe epilepsy after age and sex grouping. A 24-h day is divided into 12 2-h intervals. The x-axis represents the start time of each bin. **(A–D)** Shows the rhythm pattern of clinical seizure frequency by age and sex group, and **(E–H)** shows the rhythm pattern of clinical seizure duration.

In our analysis of enrolled patients with temporal lobe epilepsy, we categorized participants by age and gender. We observed that the peak clinical seizure frequency occurred between 21:00 and 24:00 (*p* = 0.01) in underage patients (<18 years) and between 19:00 and 22:00 (*p* = 0.002) in adult patients (18–65 years). However, an examination of the EEG data regarding the duration of clinical seizures revealed no significant biological rhythm for either the minor or adult patient groups (*p* = 1, *p* = 0.1). Furthermore, both male and female patients with temporal lobe epilepsy did not exhibit a significant circadian rhythm in their clinical seizures (*p* > 0.05) (Figure 3).

### 3.5 Circadian rhythms in temporal lobe epilepsy show significant lateralization

**Figure 4:**
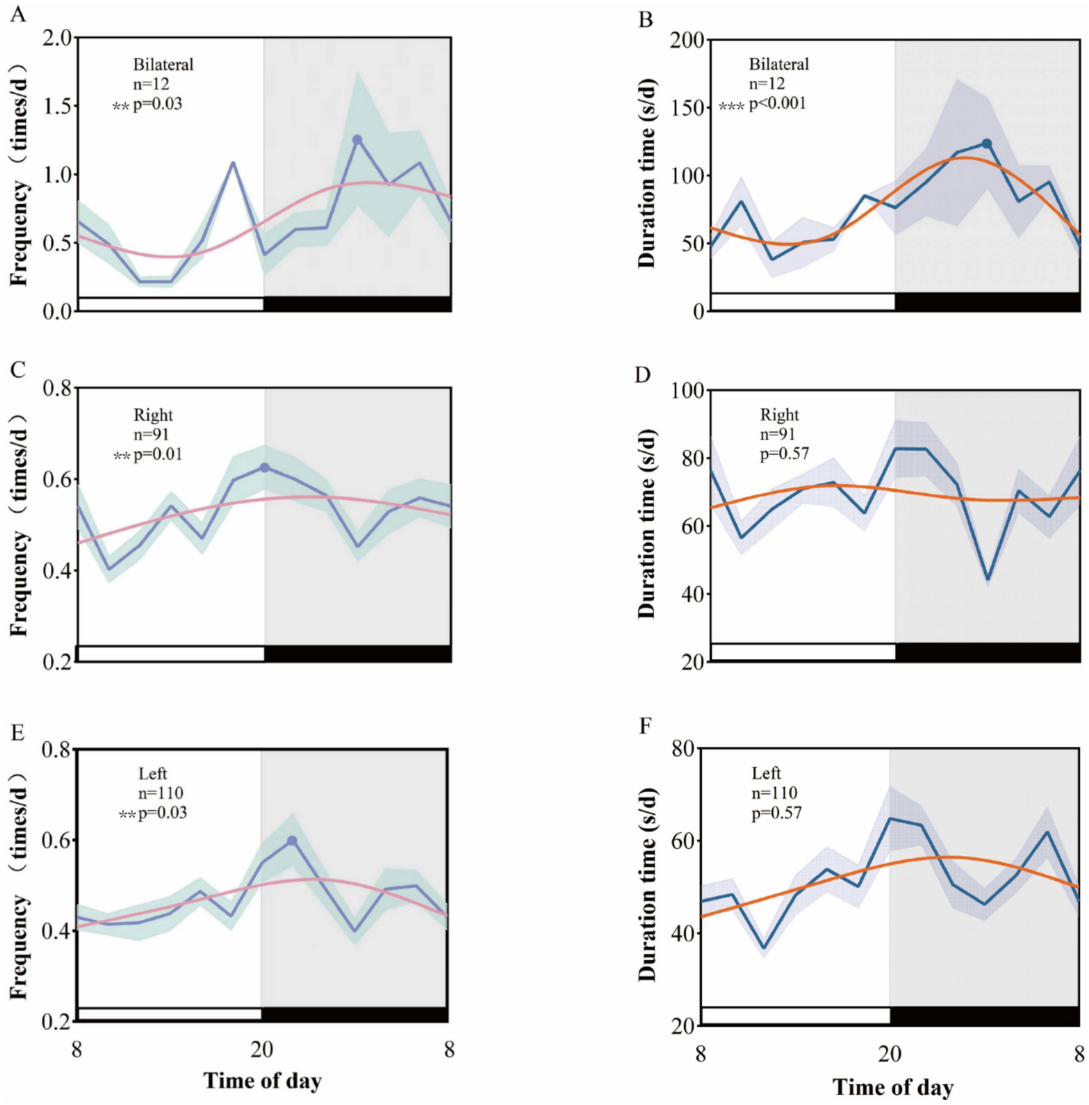
Rhythmic patterns of different laterality clinical seizures in temporal lobe epilepsy. A 24-h day is divided into 12 2-h intervals. The x-axis represents the start time of each bin. **(A)**, **(C)**, and **(E)**, respectively, show the rhythm patterns of clinical seizure frequency of left temporal lobe epilepsy, right temporal lobe epilepsy, and bilateral temporal lobe epilepsy. **(B)**, **(D)**, and **(F)**, respectively, show the rhythm patterns of left temporal lobe epilepsy, right temporal lobe epilepsy, and bilateral temporal lobe epilepsy. Rhythmic patterns of clinical seizure duration in temporal lobe epilepsy and bilateral temporal lobe epilepsy.

The analysis of EEG data from patients with varying laterality reveals that the peak clinical seizure frequency for those with left-sided temporal lobe epilepsy occurs between 19:00 and 22:00 (*p* = 0.01), whereas for right-sided temporal lobe epilepsy, the peak frequency is observed between 19:00 and 20:00 (*p* = 0.03). In contrast, neither left nor right temporal lobe epilepsy exhibited a biological rhythm in clinical seizure duration (*p* = 0.57 for both). Notably, patients with bilateral temporal lobe epilepsy demonstrated significant circadian rhythmic variations in both clinical seizure frequency (*p* = 0.03) and seizure duration (*p* < 0.001), with peaks occurring at 1:00–2:00 (Figure 4).

## 4 Discussion

This study aims to explore the diurnal pattern characteristics of clinical seizures in TLE patients across different subtypes and clinical variables. Through a retrospective analysis of 24-h video electroencephalography (VEEG) data from 213 TLE patients, we revealed that TLE patients exhibit significant circadian rhythmicity in their seizures, with distinct rhythm patterns in seizure frequency and duration observed across different subtypes, laterality, and age groups. This finding not only provides new insights into the physiological mechanisms underlying TLE but also suggests potential chronotherapy strategies for clinical treatment.

### 4.1 Circadian rhythm of seizures in TLE patients

Our research found that patients with TLE exhibit significant time-of-day rhythms in both seizure frequency and duration. Through rhythm analysis, we identified the peak times of seizures in TLE patients, especially the peak in seizure frequency. Overall, the peak in seizure frequency occurred between 19:00 and 22:00 (*p* < 0.001, *Q* < 0.001). This finding is consistent with existing studies, suggesting that seizures in TLE patients may be influenced by the circadian clock or external factors, with these influences varying across different brain regions. The circadian clock regulates cortical neural activity and the endocrine system via the suprachiasmatic nucleus (SCN) in the hypothalamus, which in turn affects the release of neurotransmitters and hormones such as cortisol and melatonin (18, 19). These factors collectively modulate neuronal excitability, potentially influencing the timing of seizures. Among external factors, the diurnal cycle induced by the Earth’s rotation is also an important determinant of the diurnal pattern (20). However, based on the current results, we are unable to determine whether the circadian rhythm of seizures in TLE patients is the result of endogenous rhythms, external cyclical factors, or a combination of both. Therefore, future research should aim to clarify the underlying mechanisms of seizure rhythm, and the causal relationship between seizure timing and endogenous versus external factors.

Despite certain limitations, our results highlight the clinical significance of seizure peak times, particularly during the 19:00–22:00 period. During hospitalization, patients were monitored with electroencephalography (EEG), and their clinical seizures may correspond to cyclical changes in factors such as the light–dark cycle, hormone secretion, sleep–wake cycles, and social activities like clinical rounds and nursing interventions. Further analysis of how these factors influence seizures in daily life is crucial for optimizing personalized treatment strategies. After establishing the overall circadian rhythm in TLE, we further investigated the rhythmic characteristics in different subtypes of TLE.

Our study found that within the same clinical subtype of TLE, the peak or trough values of seizure frequency and duration may not align. Although both seizure frequency and duration are influenced by the biological clock, they are regulated by different neurophysiological mechanisms. Seizure frequency is generally related to the excitability of neural networks and the rate of synaptic transmission (21), while seizure duration is more dependent on the stability of local brain area neural circuits and the duration of depolarization (22). Even within the same TLE subtype, the functional state of local brain regions and the coordinated activity between these regions can influence the seizure characteristics. Fluctuations in frequency may be associated with the information transmission patterns and synchrony between these regions22, whereas seizure duration may be more influenced by long-term changes in brain area electrical activity, metabolic state, and hemodynamic alterations (23). Furthermore, variations in hormone levels may also impact both seizure frequency and duration. Hormonal changes, such as those of cortisol and melatonin, have been shown to correlate with seizure activity (24). Cortisol modulates neuronal excitability by interacting with neurotransmitters (such as glutamate and GABA), thereby influencing seizure frequency (25), while melatonin affects seizure duration through its enhancement of inhibitory neural effects (26, 27). Therefore, these different mechanisms may contribute to the discrepancies in the circadian rhythms of seizure frequency and duration.

### 4.2 Seizure rhythms in different TLE subtypes

Based on the overall biological rhythm in TLE, we further investigated the seizure patterns in different TLE subtypes. According to the Kahane classification, TLE can be divided into mesial temporal lobe epilepsy (mTLE), lateral temporal lobe epilepsy (lTLE), combined mesio-lateral temporal lobe epilepsy, temporal polar epilepsy, and temporal plus. Previous studies consistently report that seizures in mTLE patients exhibit heterogeneous distribution over a 24-h period, with either a unimodal (afternoon) or bimodal (early morning and afternoon) frequency peak (7, 28–30). In this study, we found that the peak in seizure duration in mTLE occurred between 23:00 and 24:00 (*p* < 0.01), but there was no significant circadian rhythm in seizure frequency (*p* = 0.15). In contrast, lTLE patients showed no significant diurnal pattern in either seizure frequency or duration (*p* = 0.57; *p* = 1). Patients with combined mesio-lateral temporal lobe epilepsy exhibited a bimodal rhythm: seizure frequency peaked at 13:00–14:00 and 19:00–20:00 (*p* = 0.03), while seizure duration peaked at 11:00–12:00 and 19:00–20:00 (*p* = 0.01).

These results indicate significant differences in seizure rhythmic patterns across TLE subtypes, particularly in the distribution of seizure frequency and duration. This variation may be related to the different pathophysiological mechanisms, brain regions involved, and neurophysiological characteristics of each subtype. For instance, seizures in mTLE may be associated with dysfunction in the hippocampus or amygdala, while lTLE may involve more extensive cortical regions and be more influenced by external factors rather than circadian rhythm regulation.

### 4.3 The impact of age and gender on circadian rhythms

Beyond TLE subtype differences, demographic factors such as age and gender also play a crucial role in shaping seizure timing patterns. It is well established that both age and gender influence circadian rhythms in humans and animal models alike (31–33). Therefore, the potential effects of age and gender on seizure timing patterns should be considered when studying biological rhythms in seizures. Previous studies have shown that age and gender differences can influence the incidence of seizures in mesial temporal sclerosis (34). In different age groups, minors (<18 years old) had a peak seizure frequency between 21:00 and 24:00 (*p* = 0.01), while adults (18–65 years old) exhibited a peak frequency between 19:00 and 22:00 (*p* = 0.002). This suggests that age may have a significant impact on the timing of seizures in TLE patients. The peak in minors occurs later than in adults, potentially due to factors such as biological clock regulation, neurodevelopment, and hormonal levels.

With aging, the regulatory mechanisms of the biological clock may change, particularly in the phase and amplitude of core clock genes in the SCN and other brain regions (19, 35–37). These changes may explain the two-hour shift in peak seizure timing observed in adult patients compared to minors. Aging also significantly affects the neural activity rhythms of SCN cells, reducing the amplitude of these rhythms (38). Therefore, age differences may influence seizure timing in TLE patients through these biological mechanisms.

The influence of gender on circadian rhythms is complex. Although the overall analysis shows no significant differences in seizure rhythms between male and female patients (*p* > 0.05), some female patients’ seizures are linked to the menstrual cycle, exhibiting a distinct monthly rhythm (39). This suggests that hormonal fluctuations may influence seizure rhythms in some patients, particularly during different phases of the menstrual cycle. Hormonal fluctuations may alter neuronal excitability by modulating neurotransmitter systems (such as GABA and glutamate), which in turn affects seizure frequency and timing. Despite the limited overall effect of gender on seizure rhythms, future studies should further explore the potential influence of gender on different types of epilepsy, particularly in subtypes such as mesial temporal lobe epilepsy.

### 4.4 Seizure rhythms in different laterality of TLE

In addition to demographic factors, the lateralization of seizure onset further contributes to the variability in circadian seizure patterns. Specifically, we observed that patients with left temporal lobe epilepsy (LTLE) exhibited a peak in seizure frequency between 19:00 and 22:00 (*p* = 0.01), while those with right temporal lobe epilepsy (RTLE) showed an earlier peak between 19:00 and 20:00 (*p* = 0.03). These findings underscore a clear lateralization of seizure timing that may be linked to the distinct functional roles of the left and right hemispheres. The left temporal lobe, predominantly involved in language processing and cognitive tasks, tends to be more active during the day, which may explain the later seizure peak observed in LTLE patients. In contrast, the RTLE, associated with emotional regulation and spatial cognition, appears to be more influenced by circadian sleep–wake cycles and emotional factors, contributing to the earlier peak in RTLE patients.

These results suggest that the lateralization of seizure activity in TLE may reflect the differential involvement of the hemispheres in circadian rhythm regulation, with each hemisphere playing a unique role in modulating seizure timing. Further studies investigating the neurophysiological mechanisms underlying these differences—such as the roles of SCN and specific neurotransmitter systems—are needed to gain a better understanding of the complex interaction between brain hemispheres and biological rhythms in epilepsy.

### 4.5 Relationship between rhythm disruption and pathological mechanisms

This study found that TLE patients exhibit a circadian rhythm in seizure frequency, but its biological mechanisms remain to be further explored.

In terms of neuroinflammation and metabolism, animal studies have shown that diurnal pattern disruption may exacerbate neuroinflammation, leading to neuronal hyperexcitability (7), and alter brain metabolic states, affecting neural network function. Regarding hormonal regulation, cortisol influences seizure frequency by modulating the glutamate/GABA balance (25), while melatonin regulates seizure duration by enhancing inhibitory effects on the nervous system (26, 27).

In terms of clock genes, differences in the functionality of clock genes (BMAL1/CLOCK/Per/Cry) in various regions of the central nervous system can explain differences in seizure timing in focal epilepsy (20, 40). Li et al. proposed that the reduced transcription factors in the CLOCK-related epileptic brain tissue samples led to excessive excitation of pyramidal neurons, suggesting that the rhythmicity of clock genes may differ in different brain tissues (41). Abnormalities in clock genes such as PER and CRY may also disrupt brain rhythm regulation, leading to epilepsy (28). In animal experiments, the expression levels of the circadian rhythm protein Rev-erbα in the hippocampus and cortex of kainic acid-induced temporal lobe epilepsy mice peaked at night and showed a trough during the day, a result that was not observed in other brain regions, explaining the regional differences in clock gene expression (42).

Furthermore, existing research indicates that the sleep–wake cycle is strongly influenced by circadian rhythms, and seizures occurring during sleep are most likely to occur in non-REM sleep (43, 44). Deepening sleep may activate seizure-like discharges throughout the brain during the interictal period (44–46). However, because the sleep structure during the day differs from that at night, it is challenging to explain the biological rhythm of seizures by analyzing the relationship between seizures in daytime and nighttime sleep. Therefore, the underlying mechanism between the sleep–wake cycle and the circadian rhythm of seizures requires further research.

Our data were derived from patients receiving antiepileptic drug treatment (Table 3). Even though medication withdrawal or reduction was controlled during the monitoring period, drug levels fluctuate with the biological rhythm, and certain medications may alter the mechanisms of specific seizures affected by the circadian rhythm (47), thereby reducing seizures during certain periods of the circadian cycle. However, since medication withdrawal or reduction and treatment plans are individualized, the specific effects of drug levels may not be significant in subgroup analyses.

**Table 3:** Analysis of the proportional distribution of the number of ASMs taken by patients.

ASMs quantity	Counts	Proportion (%)
0	10	4.694836
1	55	25.8216
2	84	39.43662
3	47	22.06573
4	14	6.57277
5	1	0.469484
6	2	0.938967

### 4.6 Clinical implications and applications of chronotherapy

The insights gained from our investigation into seizure rhythms and their underlying mechanisms have significant clinical implications. The findings of this study lay the groundwork for the personalized treatment of TLE patients. Given the variability in seizure rhythms across TLE subtypes, patient ages, genders, and laterality, future treatment strategies may include chronotherapy, where the timing of drug administration is adjusted according to the patient’s circadian rhythm patterns to enhance efficacy and reduce side effects. For example, in patients with clear diurnal pattern, drug efficacy could be enhanced during peak seizure periods, while minimizing drug usage during periods of lower seizure frequency to optimize treatment outcomes.

Furthermore, a more comprehensive understanding of the circadian patterns in TLE will improve the design of clinical trials to evaluate the efficacy of different drugs and treatment regimens. By integrating circadian rhythm patterns into clinical trial designs, treatments can be more precisely personalized to meet the unique needs of individual patients, considering biological and clinical characteristics.

The interaction between biological rhythms and drug metabolism is particularly critical for optimizing chronotherapy. The timing of drug administration influences the absorption, distribution, metabolism, and excretion of drugs, all of which are regulated by the circadian clock. This knowledge presents an opportunity to enhance therapeutic efficacy while minimizing adverse effects. When combined with individualized circadian rhythms, chronotherapy represents a promising strategy for improving the management of TLE. Collectively, these findings underscore the potential of chronotherapy as a promising strategy for the personalized management of TLE.

### 4.7 Limitations

Although our results identified certain seizure rhythmic patterns in TLE subtypes, the sample sizes for each subtype were not consistent. Some subtypes, such as temporal polar epilepsy, may not have shown positive results due to insufficient sample size. Therefore, future studies should further investigate seizure rhythms in TLE while minimizing clinical variables. While seizures can affect circadian rhythms, circadian rhythms may also influence seizure occurrence. The mechanisms underlying this bidirectional interaction warrant further exploration.

## 5 Conclusion

This study uncovers the complex biological rhythm patterns of seizures in TLE patients, emphasizing the significant effects of clinical subtypes, age, gender, and laterality on seizure rhythms. These findings not only provide new insights into clinical treatment but also offer theoretical support for designing chronotherapy strategies. Future research should further investigate the relationship between circadian dysregulation and the underlying mechanisms of TLE seizures and validate the clinical efficacy of chronotherapy in different circadian rhythm patterns.

## 6 Future directions

Basic research should be undertaken to elucidate the mechanisms governing seizure rhythms in temporal lobe epilepsy. Specifically, studies should investigate why different TLE subtypes display divergent rhythmic patterns and whether these differences are linked to clock genes, circadian networks, or other factors. Such work will enhance our understanding of TLE seizure pathophysiology and yield clearer evidence to inform clinical diagnosis, treatment, and patient management.

## Data Availability

The original contributions presented in the study are included in the article/supplementary material, further inquiries can be directed to the corresponding authors.

## Footnotes

^1^ https://www.r-project.org/
